# Exploring the impact of a conspicuous identity on college students’ campus loans: Evidence from China

**DOI:** 10.3389/fpsyg.2022.967568

**Published:** 2022-09-07

**Authors:** Qi Zhang, Mingyang Zhang, Zhiqiang Cheng, Yiling Zhao

**Affiliations:** ^1^College of Artificial Intelligence, Nanjing Agricultural University, Nanjing, China; ^2^School of Business, Development Institute of Jiangbei New Area, Nanjing University of Information Science and Technology, Nanjing, China; ^3^School of Government, Nanjing University, Nanjing, China

**Keywords:** conspicuous identity, conformity, campus loans, college students, cognition

## Abstract

An increasing number of college students have taken out campus loans. This trend has had a negative impact on their learning and development. Using survey data about Chinese college students, this study explores the influence of a conspicuous identity and conformity on campus loan behavior and usage intention. Identify economics posits that identity is a key factor affecting individual behavior and decision-making. Differentiated identify is linked to differentiated social groups and constituted through specific value orientations, social norms and codes of conduct. This study shows that college students with a conspicuous identity are more likely to take out campus loans and more willing to take out such loans in the following year. The discussion on heterogeneity shows that a conspicuous identity has significantly positive effects on campus loan behavior for students in groups with higher consumption and grade, while conformity has significantly positive effects on campus loan behavior when students are in lower-grade groups. Compared with students who have never used campus loans, students who have taken out are more willing to take them out again in the future. Finally, avoidance strategies of campus loan debt are provided from two perspectives, namely, identity construction and the classification of education.

## Introduction

The rapid development of mobile and 5G internet technology has caused shockwaves in the consumption patterns of Chinese college students. What these students consume has become more varied, and their consumption patterns have been transformed and upgraded (Sheng and Xiao, 2016; Lin and Li, 2021). Data from iiMedia Research shows that, in 2021, the annual consumption of Chinese college students is expected to exceed ¥700 billion (iiMedia Research, 2021). However, their consumption is constrained by income. More than 90% of college students in China depend completely on their parents’ finances. To satisfy students’ diversified demand, credit consumption has developed greatly on college campuses (Zhu, 2019). In China, campus loans are roughly divided into three types: installment shopping platforms, such as Qudian and Fenqile; P2P loan platforms, such as Toutoudai and Mingxiaodai; and consumer credit services supplied by e-commerce platforms, such as Jd.baitiao and Ant Credit Pay. The Nandu Poll Centre found that 12.39% of college students have used installment shopping platforms, 7.02% have used P2P loan platforms, and 44.57% have used consumer credit services supplied by e-commerce platforms (Nandu Poll Centre, 2017). However, the use of campus loans leads to a series of social problems due to lending non-compliance procedures. Frequent and excessive use of campus loans will make students develop incorrect concepts of consumption and create mind of rivalry. Some students may experience great symptoms of psychological distress due to failure to repay in time, affecting their physical and mental health and learning performance. And some ones in personal debt crisis, even repay the loan by taking out loans on another platform. These problems have brought great challenges for colleges and universities to help college students overcome mind of rivalry, and cultivate correct concepts of consumption and financial management. In 2017, the China Banking and Insurance Regulatory Commission, the Ministry of Education of the People’s Republic of China, and the Ministry of Human Resources and Social Security of the People’s Republic of China issued a joint *Notice on Further Strengthening the Regulation of Campus Loans*, which requested that network loan institutions suspend the network loan business for college students. However, some network lending platforms have continued to offer loans to students by reinventing themselves.

College students are in transition to adulthood, and their self-identity faces uncertainty. Driven by live celebrity broadcast (such as Taobao live, Douyin live, etc.) and shopping sharing platforms (such as Xiaohongshu APP and Miya APP), college students yearn for the “exquisite” life hyped in the media. New consumption patterns, such as conformity consumption, conspicuous consumption and brand consumption, have appeared on college campuses (Wang et al., 2010; Zhu, 2014). College students may be more inclined to borrow money for consumption due to conspicuous or conformity consumption. Zhang (2009) has highlighted that conspicuous consumption may make consumers converge to specific identities. Under these circumstances, this study, which is both theoretically and practically significant, examines the heterogeneous impact of conspicuous identity and conformity on campus loan behavior and usage intention.

The importance of college student loans and credit card debt have been of increasing concern. The 2018 Report on the Economic Well-Being of United States Households shows that United States student loans are held by 93% of those who have outstanding education debt, while, 24% of those who have such outstanding debt borrowed against credit cards (Board of governors of the federal reserve system, 2018). The literature has shown that campus loans have brought about a series of social problems. For instance, credit card loans are associated with wide-ranging adverse health indicators in college students (Nelson et al., 2008), force students to reject or significantly delay marriage (Haneman, 2017), hinder entrepreneurship (Krishnan and Wang, 2019), and influence their full-time employment upon graduation (Froidevaux et al., 2020). Some studies have explored the factors leading to credit card debt. Brougham et al. (2011) found that compulsive buying significantly increases college students’ credit card debt. Wang and Xiao (2009) similarly found that impulse buying and social networks lead college students to accumulate credit card debt. Fagerstrøm and Hantula (2013) found that, in the internet era, consumer behavior may be related to innovative payment methods. College students who don’t usually use a credit card don’t opt to purchase a phone immediately on credit but prefer to delay purchase until the money was saved. Meyll and Walter (2019) found that individuals who use their smartphones to conduct mobile payments are more likely to exhibit costly credit card behavior. Zhu et al. (2020) found that online shopping was the most significant predictor of credit consumption. The literature has focused on the social problems caused by campus loans and has explored not only contributing psychological factors (such as compulsive and impulse purchases), but also online shopping and innovative payment methods. However, there has been little research on the impact of identity on campus loan behavior.

In this study, we explore the influence of a conspicuous identity and conformity on campus loan behavior and usage intention. The results show that college students with a conspicuous identity are more likely to take out campus loans and more willing to take them out in the following year. College students with a conspicuous identity seek to leave ordinary groups on campus, hoping to gain admiration from other groups through their unconventional and high consumption levels. A conspicuous identity has a significantly positive impact on campus loan behavior for students in higher consumption and grade groups. Conformity has a significantly positive impact on campus loan behavior for students in lower grade groups. In the early stage of college life, students are in the adaptable stage. They are eager to be integrated into a specific campus group to fulfill their needs of belonging. They may convey convergence signals to a specifically desired campus group through daily consumption. As they move up through the grades, some students may pursue innovative, creative and distinctive consumption items with ostentatious character, in order to set themselves apart from other campus groups. However, constrained by economic condition, they have to use campus loans to relieve the pressure this consumption causes. Otherwise, compared with students who have never taken out campus loans, students who have taken them out before are more willing to take them out again in the future. Otherwise, compared with students who have never taken out campus loans, students who have taken them out before are more willing to take them out again in the future.

## Theoretical framework and hypotheses

Identity economics posits that identity is a key factor affecting individual behavior and decision-making, and differentiated identities bring about differentiated decision-making and behaviors (Li and Xu, 2016). Differentiated identity is linked to differentiated social groups and constituted through specific value orientations, social norms and codes of conduct. These values and norms affect individuals’ behaviors and decision-making. The study of identity originated in philosophy and developed in psychology. Tajfel et al. (1971) proposed that individual identity is developed when an individual identifies role for himself or herself in relation to values, beliefs, norms and goals. Akerlof and Kranton (2000) incorporated the psychology and sociology of identity into a behavioral economic model and considered how identity, i.e., a person’s sense of self, affects economic outcomes. In view of this, different identities may deeply influence college students’ behaviors, including the decision to take out campus loans.

In *Consumer Society*, displaying similarities and differences through consumption is also about constructing heterogeneous identities. Baudrillard (2001) indicated that commodities are valued for their symbolic meanings rather than their use; in many cases, only the meanings are consumed. He proposed that people use material objects to symbolize their identity in a positive light to either join ideal groups or leave them to join higher status groups. During such a life transition stage, students’ ideological cognition is uncertain. College students’ identities and status are intertwined. Influenced by multiculturalism, college students consciously consider specific class cultures and identities and show appreciation and subservience (Zhang, 2019). When the identity of their surroundings is uncertain, college students use their daily consumption behavior to show an appreciation for specific classes on campus and in turn construct their identity. When college students consume, similarities and differences are communicated as signals. Students understand their self-identity again, identify themselves with a group, or abandon a group to join another (Chen and Zhang, 2018). When students display their similarities, they highlight sameness, consistency and unity with the social class with which they identify. Conformity is paired with the phenomenon that occurs when people display similarities in that people consciously and indiscriminately imitate the actions, thoughts, and expressions of people belonging to a certain group to obtain its approval and join it. People buy the same brands of clothing, cosmetics, electronics, etc., as others in the group, and feel uncomfortable while they don’t have those items. People use consumption to highlight difference in social class. A conspicuous identity is paired with the phenomenon of displaying differences, in that people attempt to distinguish themselves from others and show social identity through higher levels of food, clothing, housing and transportation consumption, as well as through flashier consumption. Veblen and Mills (2017) contended that the leisure class influences lower-status groups via conspicuous consumption, which results in those groups in turn engaging in conspicuous consumption. A conspicuous identity represents the partial alienation of conformity that college students seek and the re-encoding and removal of their respective individual social identities.

Consequently, when college students attempt to build conspicuous identities or conformity, they use clothing, food, housing and transportation consumption to show a convergence toward the cultural symbols of specific classes on campus, and then integrate into the identity group with which they identify. However, college students are restricted by their budget, and their daily consumption mainly relies on their parents’ support. The additional expenditure triggered by their identity seeking may prompt some college students to take out campus loans to meet their consumption needs. This study proposes the hypothesis that college students with a conspicuous identity or conformity are more likely to take out campus loans.

## Data and methods

### Data

The survey included questions regarding conspicuous identity, conformity, credit behavior, and sociodemographic status. Experts evaluated the reliability of such a self-administered questionnaire. The preliminary questionnaire was revised after a preliminary survey was conducted. The questionnaire consisted of 4 parts and a total of 28 questions. Refer to the existing literatures, conspicuous identity was evaluated by a five-item scale: ① I feel good about spending money in front of my classmates (Meng, 2016); ② I like buying new and fashionable goods that can reflect my quality of life and taste (Meng, 2016); ③ I am eager to show my social worth and status through high expenditures on food, clothing, shelter and transportation (Charles et al., 2009; Verdugo et al., 2020); ④ I hope that when I buy something, other students will enthusiastically buy it too; ⑤ I often buy products that have no use value but that give me confidence and make me proud (Bronner and de Hoog, 2018).

According to the relevant studies, the role of conformity was also measured by a five-item scale: ① I associate with students who have the same levels of consumption as mine; ② My good friends are those who share my purchase and consumption habits, for example in the choice for brands of clothing, cosmetics, mobile phones, etc.; ③ Conformity mentality takes over in my buying decision process; ④ I compare my consumption behavior to that of my classmates’; ⑤ I feel ashamed and fear being looked down upon when I do not have goods my classmates have, for example phones, computers and watches of a certain brand (Kang and Ma, 2020). Respondents evaluated the abovementioned two five-item scales using a nine-point Likert-type scale where 1 means completely disagree, while 9 means completely agree.

College students’ credit behavior involves attitude, behavior, and willingness to take out campus loans. It also involves loan sources and purposes, and repayment method. Sociodemographic status includes the type of university, gender, grade, household disposable income, personal monthly consumption, parents’ education level, etc.

An online survey was conducted via the *Wenjuanxing* platform over the period ranging from February 27, 2021, to March 7, 2021; the *Wenjuanxing* platform is a professional online questionnaire, evaluation and voting platform in China. This platform has had paying customers in more than 30,000 companies and in 90% of Chinese universities. It has issued 148 million questionnaires and received 11.744 billion answers. It has been widely used by Chinese scholars to carry out academic research, administer social surveys and collect information. For instance, the China Youth Research Centre used the *Wenjuanxing* platform to conduct an investigation about teenagers’ working conditions (Wang Y. X. et al., 2021). Wang Y. et al. (2021) and Suo et al. (2022) also used the platform to collect data for their exploration of individuals’ behavior or intention. This study used the self-administered survey. Respondents conducted the questionnaire by himself or herself via *Wenjuanxing* platform. So, some respondents may not fill in carefully. On the contrary, some ones may fill in carefully, and they may search more information online which may change their cognition. These issues may affect our results. Designing the questionnaire online, we made some efforts to mitigate those problems. For instance, the validity of the questionnaire was judged through time to complete the questionnaire. If the time is less than 2 minutes or more than 10 minutes, it was regarded an invalid questionnaire. We also set some trap items. For example, the respondents were asked to respond the question that what is your gender every ten items. We have changed the order of choices to judge the valid of questionnaire.

A total of 581 valid questionnaires were received. College students from the world’s “first-class universities and disciplines” (“double tops”) accounted for 31.15% of the respondents. The Central Committee of the Communist Party and The State Council of China have developed “double tops” as a national higher education strategy. Aside from the “double tops” students, respondents included students from ordinary undergraduate institutions, who accounted for 57.49% of all the respondents, while 11.36% came from junior colleges. Among the respondents, 271 were male, accounting for 46.64%. Freshmen, sophomores, juniors, seniors or above accounted for 19.97, 31.67, 32.87, and 15.49%, respectively. The household disposable income showed great variation, with the categories of less than *¥10,000, ¥10,000–30,000, ¥30,000–50,000, ¥50,000–1,00,000, ¥1,00,000–2,00,000*, and more than *¥2,00,000* accounting for 16.52, 25.13, 9.64, 20.83, 16.35, and 11.53%, respectively. Students spending *¥800–1,500* a month accounted for the highest proportion, at 65.23%. Students with parents with a secondary education accounted for 33.05%, followed by those with parents with a high school education at 22.72%. Students with parents who had earned a bachelor’s degree accounted for 18.59% of the respondents.

### Methods

To better quantify the impact of different types of identity on college students’ borrowing behavior, a binary logit model is used. The logit model has been widely used to analyze individual behavior. For instance, Zhang C. H. et al. (2021) constructed a binary logit model to explore the factors influencing college students’ consumption tendency. And they set a dummy variable to measure college students’ tendency of taking microloan. 1 means someone took loans from online platform. Use a binary logit model, Qian et al. (2019) analyzed the influence of urban identity on transfer of the right to use rural homestead. But in their model, urban identity was measured by a dummy variable. The following empirical model is specified.


(1) $$Y_{i}=\beta_{0}+\beta_{1}\,C\,o\,n\,f\,o\,r\,m\,i\,t\,y_{i}+\beta_{2}\,C\,o\,n\,s\,p\,i\,c\,u\,o\,u\,s_{i}+f\,\left(X_{i};\delta \right)+\mu_{i}$$

Here, β and δ are the parameters of the independent variables to be estimated, while μ is a random disturbance term. The explained variable *Y*_*i*_ describes whether the *i*–thcollege student has ever taken out campus loans, for example through installment shopping or P2P loan platform or consumer credit services supplied by e-commerce platforms. Refer to the theoretical analysis in this manuscript, we set the explanatory variables according to the theory of *Consumer Society.* Heterogeneous identities can be constructed from two aspects, including displaying similarities and differences through consumption. A conspicuous identity and conformity are used to reflect the phenomenon of displaying differences and similarities, respectively. Conspicuous identity and conformity are evaluated by a five-item scale.

Some studies have stated that household disposable income, monthly consumption (Han et al., 2015), grade, gender, student’s place of origin (Pang and Cheng, 2017; Wang and Zhou, 2017), qualifications, major (Zhou et al., 2019),parents’ education level, ethnicity, debt aversion level (De Gayardon et al., 2022), and levels of financial literacy (Artavanis and Karra, 2020) are important in influencing not only credit card debt but also student and campus loans. In this study, multiple sociodemographic characteristics (*X*i**) are included in the model. The type of university is measured by four dummy variables, namely, *first-class university, first-class discipline, ordinary undergraduate institution, and junior college*. The “first-class” classification is used as the base category. Students’ grade is measured by four dummy variables: *freshmen* (base), *sophomores*, *juniors*, *seniors* or above. Students’ discipline is measured by five dummy variables: *agriculture or medical science (base)*, *economics and management, humanities and social sciences, science and engineering*, and *arts.* Family disposable income is measured by five dummy variables: *more than ¥2,00,000*, *¥1,00,000–2,00,000, ¥50,000–1,00,000 (base), ¥ 30,000–5,0,000*, and *less than ¥30,000*. *Male* is both a dummy variable and the base category. Monthly consumption expenditure is measured by five dummy variables: *less than ¥800 (base), ¥800–1,200, ¥1,200–1,500, ¥1,500–2,000*, and *more than ¥2,000.* The education level of parents is measured by four dummy variables: *junior high school or below, high school or secondary technical school, junior college*, and *bachelor’s degree or above* (see Table 1).

**TABLE 1:** Dependent and independent variables with their definitions and descriptions.

Variable	Description and measurement	Mean	Standard deviation
**Explained variables**			
*Borrowing behavior with regard to campus loans*	Whether college student has ever used a campus loan, such as installment shopping platform or P2P loan platform or consumer credit service supplied by an e-commerce platform. (1 = yes; 0 = no)	0.559	0.497
*Usage intention of campus loans*	Do you intend to use campus loans in the following year? (1 = willing; 0 = unwilling)	0.353	0.478
**Explanatory variables**			
*Conspicuous identity*	Conspicuous identity was evaluated by a five-item scale. (1 = completely disagree; …; 9 = completely agree)	3.221	1.702
*Conformity*	Conformity was evaluated by a five-item scale. (1 = completely disagree; …; 9 = completely agree)	3.890	1.492
**Sociodemographic characteristics**			
Type of university	What type of university do you attend?		
*first-class university(base)*	1 = yes; 0 = no	0.157	0.364
*first-class discipline*	1 = yes; 0 = no	0.155	0.362
*Ordinary undergraduate institution*	1 = yes; 0 = no	0.575	0.495
*Junior college*	1 = yes; 0 = no	0.114	0.318
Discipline	What is your major?		
*Agriculture or medical science(base)*	1 = yes; 0 = otherwise	0.129	0.336
*Economics and management*	1 = yes; 0 = otherwise	0.256	0.437
*Humanities and social sciences*	1 = yes; 0 = otherwise	0.158	0.365
*Science and engineering*	1 = yes; 0 = otherwise	0.425	0.495
*Arts*	1 = yes; 0 = otherwise	0.031	0.173
Grade	What grade are you in?		
*Freshmen (base)*	1 = yes; 0 = otherwise	0.200	0.400
*Sophomores*	1 = yes; 0 = otherwise	0.317	0.466
*Juniors*	1 = yes; 0 = otherwise	0.329	0.470
*Seniors or above*	1 = yes; 0 = otherwise	0.155	0.362
**Family disposable income**			
*More than ¥2,00,000*	1 = yes; 0 = otherwise	0.115	0.320
*¥1,00,000–2,00,000*	1 = yes; 0 = otherwise	0.164	0.370
*¥50,000–1,00,000(base)*	1 = yes; 0 = otherwise	0.208	0.406
*¥ 30,000–5,00,000*	1 = yes; 0 = otherwise	0.096	0.295
*Less than ¥30,000*	1 = yes; 0 = otherwise	0.417	0.493
*Male*	1 = male; 0 = female	0.466	0.499
**Monthly consumption expenditure**			
*Less than ¥800 (base)*	1 = yes; 0 = otherwise	0.105	0.307
*¥800–1,200*	1 = yes; 0 = otherwise	0.351	0.478
*¥1,200–1,500*	1 = yes; 0 = otherwise	0.301	0.459
*¥1,500–2,000*	1 = yes; 0 = otherwise	0.133	0.339
*More than ¥2,000*	1 = yes; 0 = otherwise	0.110	0.313
Education level of parents	What is the education level of your parents?		
*Junior high school or below* (base)	1 = yes; 0 = otherwise	0.422	0.494
*High school or secondary technical school*	1 = yes; 0 = otherwise	0.270	0.444
*Junior college*	1 = yes; 0 = otherwise	0.110	0.313
*Bachelor degree or above*	1 = yes; 0 = otherwise	0.198	0.399

## Results

### Descriptive statistics

A statistical summary of the variables is presented in Table 1. For all variables, the mean values are greater than 0, and the standard deviation is small. Although 73.15% of the respondents did not agree about using campus loans for excess consumption, 55.94% had used campus loans. This result reflects that while most college students can rationally realize the disadvantages of excess consumption, there is a conflict between the desire to consume and the desire to restrain consumption. This conflict leads some students to be unable to stop using campus loans. Additionally, 35.28% of the respondents intended to use campus loans in the following year, which is significantly lower than the current use rate. This further indicates that there is inconsistency between cognition and behavior among college students. This result echoes survey results obtained by Liu (2018).

The survey also shows that Ant Credit Pay has become the preferred lending platform for college students, and 88.92% of respondents have used it. This is followed by *Jd.baitiao* and *Fenqile*, with 3.69 and 2.15%, respectively. Because most college students do not have a stable income, Chinese banks rarely issue credit cards to college students; therefore, only 1.54% of them reported having credit cards. When questioned about the purpose of the loan, 59.69% of the college students said they used it for shopping, while only 8% used it for training and study. Moreover, 65.54% of college students reported repaying their campus loans by working part-time jobs, and 17.23% reported choosing to repay their loans by borrowing from family and friends. In contrast, 17.23% reported repaying the loan by taking out loans on another platform.

### Benchmark results

Table 2 reports the estimated results from the logit model regarding the influence of a conspicuous identity and conformity on campus loan-taking behavior. Based on the values of means and standard deviation, the observed values of each variable satisfy the hypothesis of non-homogeneity. The value of variance inflation factor (VIF) is equal to 1.81 (less than 5) indicating that there is no serious problem with multicollinearity between the variables selected by the model. The condition number is 20.45 which is less than 30 and indicates no concern of multicollinearity. The Wald test (*p* < 0.001) suggests the logit model with robust standard errors provides valid estimation results. The factors listed in Column (1) of Table 2 are not controlled, while in Column (2), they are controlled. The results indicate that the marginal effect of a conspicuous identity is overestimated when control variables are not omitted (0.248 > 0.222). This study develops an estimation model using cluster-robust standard error (see Column (3) in Table 2) to reduce the possible heteroskedasticity. The results show that the ordinary standard errors of most variables are similar to the cluster standard errors, which indicates that the benchmark model constructed in this study does not have a serious problem with heteroscedasticity. According to the results shown in Column (2), the study calculates the marginal effects of variables (see Column (4) in Table 2).

**TABLE 2:** The estimation results of campus loan behavior.

Variables	(1)	(2)	(3)	(4)
	Non-controlling for other factors	Using ordinary standard error	Using robust standard error	Marginal effects after (2) regress
*Conspicuous identity*	0.248***	0.222***	0.222***	0.055***
	(0.073)	(0.081)	(0.080)	(0.019)
*Conformity*	0.013	0.060	0.060	0.015
	(0.081)	(0.089)	(0.086)	(0.022)
*first-class disciplines*		0.598*	0.598*	0.141*
		(0.332)	(0.329)	(0.073)
*Ordinary undergraduate institutions*		0.903***	0.903***	0.219***
		(0.271)	(0.275)	(0.064)
*Junior college*		0.826**	0.826**	0.188**
		(0.373)	(0.375)	(0.075)
*Economics and management*		0.564*	0.564*	0.135*
		(0.318)	(0.322)	(0.073)
*Humanities and social sciences*		0.339	0.339	0.082
		(0.348)	(0.356)	(0.082)
*Science and engineering*		0.270	0.270	0.066
		(0.301)	(0.309)	(0.073)
*Arts*		0.936	0.936	0.204
		(0.608)	(0.591)	(0.110)
*Sophomores*		0.233	0.233	0.056
		(0.260)	(0.258)	(0.063)
*Juniors*		1.065***	1.065***	0.248***
		(0.269)	(0.267)	(0.058)
*Seniors*		1.303***	1.303***	0.280***
		(0.330)	(0.327)	(0.058)
*Male*		0.147	0.147	0.036
		(0.205)	(0.205)	(0.050)
*more than ¥2,00,000*		–0.191	–0.191	–0.047
		(0.379)	(0.375)	(0.094)
*¥1,00,000–2,00,000*		–0.790**	–0.790**	–0.194**
		(0.316)	(0.307)	(0.076)
*¥ 30,000–5,00,000*		–0.192	–0.192	–0.048
		(0.354)	(0.355)	(0.088)
*less than ¥30,000*		–0.236	–0.236	–0.058
		(0.254)	(0.253)	(0.063)
*¥800–1,200*		0.904***	0.904***	0.214***
		(0.330)	(0.336)	(0.074)
*¥1,200–1,500*		1.358***	1.358***	0.2307***
		(0.344)	(0.349)	(0.074)
*¥1,500–2,000*		1.246***	1.246***	0.268***
		(0.404)	(0.405)	(0.071)
*more than ¥2,000*		1.213***	1.213***	0.260***
		(0.454)	(0.457)	(0.078)
*High school or secondary technical school*		–0.014	–0.014	–0.004
		(0.231)	(0.231)	(0.057)
*Junior college*		0.092	0.092	0.023
		(0.327)	(0.326)	(0.080)
*Bachelor degree or above*		0.020	0.020	0.005
		(0.282)	(0.271)	(0.069)
Constant	–0.594**	–3.189***	–3.189***	
	(0.241)	(0.587)	(0.619)	
Observations	581	581	581	581
*R*-squared	0.031	0.117	0.117	0.117

Robust standard errors in parentheses, ***p < 0.01, **p < 0.05, *p < 0.1.

The marginal effect of *conspicuous identity* is 0.055 and significant. This result shows that the probability of using campus loans increases by 5.5% for each 1 unit increase in the recognition of a conspicuous identity. However, the estimated coefficient of *conformity* is not significant, which indicates that conformity has no effect on the campus loan-taking behavior of college students. The estimated coefficients of *first-class disciplines, ordinary undergraduate institutions*, and *junior colleges* are 0.141, 0.219, and 0.188, respectively; they are also significant. These results show that, compared with first-class universities students, college students from first-class disciplines, ordinary undergraduate institutions and junior colleges are more likely to use campus loans. The estimated coefficients of *juniors*, seniors or above are significantly positive, which indicates that, compared with freshmen, juniors, seniors or above are more likely to use campus loans. The estimated coefficient of household disposable income *¥1,00,000–2,00,000* is significantly negative. These results indicate that compared with the base group (*¥50,000–1,00,000*), college students with a household disposable income of *¥1,00,000–2,00,000* show a lower probability of using campus loans. The estimated coefficients of monthly consumption for the ranges of *¥800–1,200*, *¥1,200–1,500*, *¥1,500–2,000*, and *more than ¥2,000* are significantly positive. These results indicate that compared with the base group (*less than ¥800*), college students with higher consumption levels are more likely to use campus loans. Studying economics and management has a significant and positive impact on campus loan behavior. The results show that students majoring in economics and management are more likely to take out campus loans than students in other majors.

### Discussions on heterogeneity: Grouped according to monthly consumption

The results of the benchmark model (see Table 2) show that monthly consumption has a significantly positive impact on campus loan behavior. The volume of college students’ monthly consumption varies greatly. According to the survey data, respondents whose monthly consumption is less than ¥1,200, ¥1,200–1,500, ¥1,500–2,000, and more than ¥2,000 account for 45.61, 30.12, 13.25, and 11.02%, respectively. The above monthly consumption levels are divided into two groups, namely, a lower consumption group (with a monthly consumption of less than ¥1200) and a higher consumption group (with a monthly consumption of more than ¥1,200). The results (see Table 3) indicate that a conspicuous identity has significantly positive effects on campus loan behavior in the higher consumption group. However, the effect of a conspicuous identity on campus loan behavior in the lower consumption group is not significant. The results show that when driven by a conspicuous identity, college students with a higher monthly consumption are more likely to take out campus loans. There is no significant heterogeneity in the influence of conformity on campus loan behavior in college students across the lower and higher consumption groups.

**TABLE 3:** The estimation results of campus loan behavior grouped by monthly consumption and grade.

Variables	(1)	(2)	(3)	(4)
	Lower consumption group	Higher consumption group	Lower grade	Higher grade
*Conspicuous identity*	0.169	0.076***	0.019	0.091***
	(0.132)	(0.026)	(0.027)	(0.027)
*Conformity*	0.126	–0.010	0.062**	–0.044
	(0.141)	(0.028)	(0.030)	(0.029)
Control variables	Yes	Yes	Yes	Yes
Observations	265	316	300	281

Robust standard errors in parentheses, ***p < 0.01, **p < 0.05, *p < 0.1.

### Discussions on heterogeneity: Grouped according to grade

The results of the benchmark model (see Table 2) show that grade has a significantly positive impact on campus loan behavior. These results echo findings by Hao et al. (2019) and Baum (2016). The survey results show that freshmen, sophomores, juniors, seniors or above account for 19.97, 31.67, 32.87, and 15.49%, respectively (in China, undergraduate majors such as medicine usually have a five-year curriculum). In this study, the abovementioned grades are divided into two groups, namely, a lower grade (freshman and sophomore) and a higher grade (junior and above). The results (see Table 3) indicate that conformity has significantly positive effects on campus loan behavior in the lower grade group, while a conspicuous identity significantly affects campus loan behavior in higher grade students. The survey results show that the proportion of higher grade students using campus loans is 67.85%, which is 23% higher than the proportion of lower grade students. Conspicuous consumption may be the main incentive for campus loan behavior in higher grade students.

### Extended discussion

To grasp the trend in campus loans, this study analyzes the impacts of campus loan behavior, conformity, and a conspicuous identity on college students’ usage intention of campus loans in the following year. A binary model was developed to estimate this influence because the usage intention of campus loans is a binary variable. The following empirical model is specified:


(2) $$I\,n\,t\,e\,n\,t\,i\,o\,n_{i}=\beta_{0}+\beta_{1}\,C\,o\,n\,f\,o\,r\,m\,i\,t\,y_{i}+\beta_{2}\,\text{C}\,o\,n\,s\,p\,i\,c\,u\,o\,u\,s_{i}+\varphi \,Y_{i}+f\,\left(X_{i};\alpha \right)+\varepsilon_{i}$$

Here β , φ and α are the parameters of the independent variables to be estimated, while ε is a random disturbance term. The explained variable *Intention*_*i*_ indicates whether the *i*−*th* college student is willing to take out campus loans in the following year. The control variables are the same as those in Equation (1).

Table 4 reports the estimated results. The marginal effect of campus loan behavior is 0.351 and significant. The results show that, compared with students who have never used campus loans, the probability of usage intention of campus loans increases by 0.351 for those students who have used campus loans. The estimated coefficient of *conspicuous identity* is significant and positive, which indicates that the probability of usage intention of campus loans increases by 0.039 for each 1 unit increase in the recognition of a conspicuous identity. However, the estimated coefficient of *conformity* is not significant.

**TABLE 4:** The estimation results of campus loan intention.

Variables	(1)	(2)	(3)	(4)
	Non-controlling for other factors	Using ordinary standard error	Using robust standard error	Marginal effects after (2) regress
*Campus loan behavior*	1.745***	1.769***	1.769***	0.351***
	(0.212)	(0.230)	(0.232)	(0.039)
*Conspicuous identity*	0.195**	0.185**	0.185**	0.039**
	(0.079)	(0.085)	(0.086)	(0.018)
*Conformity*	0.002	0.002	0.002	0.000
	(0.089)	(0.094)	(0.096)	(0.020)
Control variables	Yes	Yes	Yes	Yes
Constant	–2.368***	–2.859***	–2.859***	
	(0.301)	(0.644)	(0.628)	
Observations	581	581	581	581
*R*-squared	0.139	0.175	0.175	

Robust standard errors in parentheses, ***p < 0.01, **p < 0.05, *p < 0.1.

## Discussion

The empirical results show that a conspicuous identity has a significant impact on both college students’ campus loan behavior and usage intention. College students with a conspicuous identity seek to leave ordinary groups on campus, hoping to gain admiration from other groups through their unconventional and high consumption levels. Driven by modern media technology, some college students equate high consumption levels with high status and top brands with quality of life in their effort to transcend their social class at school (Zhu and Zhang, 2019). Therefore, a conspicuous identity is more likely to stimulate the occurrence of campus loan behavior and enhance students’ intention to take out those loans. The results echo findings by Charles et al. (2009) and Verdugo et al. (2020). They found that personality traits could influence young adults’ conspicuous consumption. For instance, they are eager to show their social status through conspicuous consumption, such vacation and brand clothing. Bronner and de Hoog (2018) also got the similar results that conspicuous consumption not only means the ostentation of wealth but also the demonstration of something symbolic that is more immaterial. Campus loans provide a convenient and satisfactory tool for college students to engage in conspicuous consumption and borrow money from lending institutions based on low threshold characteristics, fast borrowing and more receivables (Wang and Xiao, 2009; Cao, 2020).

The results of the heterogeneity discussion grouped by grade show that conformity has a significantly positive impact on campus loan behavior in lower grade students, while a conspicuous identity has a significant and positive influence on campus loan behavior in higher grade students. In the early stage of college life, young students coming from different regions and social groups, with specific identities and cultures, get together (Chen and Zhang, 2018). At the same time, college students who are in the adaptable stage of college life are eager to be integrated into a specific campus group to fulfill their needs of belonging (Zhang et al., 2018). Therefore, college students may convey convergence signals to a specifically desired campus group through daily consumption. When this is the case, the consumption pattern usually follows the conformity trend. The results echo findings by Kang and Ma (2020). They found teenagers often wear the same brand jacket. As a psychological trait, the fear of missing out can describe why young adults want to belong to a main group. Zhang (2022) also found similar findings that conformity psychology is a major factor influencing people to buy goods. College students represent a young group who is extremely concerned with the internet and seek out novelty and fashion. As they move up through the grades, college students gradually appear to be well integrated into campus life. Driven by the internet and internet celebrity economy, some students may be influenced by social media information about consumption and fashion (Fu et al., 2020). They then may pursue innovative, creative and distinctive consumption items with ostentatious character, in order to set themselves apart from other campus groups. Once they adopt the consumption psychology of keeping up with others, they have higher living standard requirements (e.g., for electronic products, cosmetics, or clothing; Zhang C. et al., 2021). However, because they are constrained by their economic condition, whether they engage in follow-up or conspicuous consumptions, college students must use campus loans to relieve the pressure this consumption causes.

Additionally, monthly consumption has a significant impact on campus loan behavior, and the marginal effect of a conspicuous identity is also positive for the higher consumption group. This result aligns with the findings by Lachance (2012). With the rapid development of China’s economy, college students’ consumption behavior is more diversified, and they pay more attention to their psychological needs. On the one hand, what students consume is increasingly rich, including in the areas of food, education, leisure and entertainment, interpersonal items, clothing, communication, romantic items, tourism, and so on (Frick et al., 2021). On the other hand, the consumption structure has been transformed and upgraded, and consumers’ focus has turned from functionality to product quality, brand and appearance. Chinese college students mainly depend on their parents’ finance. The increase in their monthly consumption levels may cause economic pressure on college students and induce them to take out campus loans. The current study indicates that students with higher monthly consumption are more likely to take out campus loans.

## Conclusions and implications

Using survey data about Chinese college students, this study explores the impacts of conspicuous identity and conformity on campus loan behavior and usage intention. The study shows that college students with a conspicuous identity are more likely to take out campus loans and be more willing to take them out again in the following year. The discussion on heterogeneity shows that a conspicuous identity has significantly positive impacts on campus loan behavior in students from higher consumption groups and higher-grade groups, while conformity has significantly positive impacts on campus loan behavior in students from lower grade groups. Compared with students who have never used campus loans, students who have used them are more willingness to take them out again in the future.

The results have the following value. The factors affecting campus loan behavior are complex, including both subjective and objective aspects. This study expands the theory of identity economics by constructing a theoretical framework analyzing the impact of conspicuous identity and conformity on campus loan behavior and usage intention. This study also deepens the understanding of the motivation and nature of college students’ campus loan behavior. This research framework can be extended to other countries or regions with rapid economic development but incomplete social credit systems. This study also discusses how to eliminate the adverse effects of a conspicuous identity, thus providing a reference for decision-makers in the fields of education and management to protect college students from falling into campus loan debt.

Finally, this manuscript provides some strategies for effectively avoiding campus loan debt from two perspectives of identity construction and various teaching. First, education management departments should guide college students to build fitting identity and establish a positive outlook on life. We should alert students and strongly signify how harmful campus loan debt is by publicizing the cases of college students who are deep into campus loan debt. We should also strengthen our teaching about what to expect from life, taking into account college students’ aspirations for a better life, and encouraging them to realize the value of life and how to achieve a happy life through personal struggles. We should guide students not only to have dreams, but also to achieve those dreams by working hard and realizing the richness of the spiritual world and material life. Second, education management departments should focus on graded guidance and classified implementation of policies and improve the relevance of education. We should teach seniors about finances, with a focus on identifying credit loan platforms, so that students can avoid campus loan traps. We should also teach them about financial management to cultivate a correct attitude toward money. For lower grade students, we should focus on fostering different conceptions of consumption, and educate them about how to live day to day, and we should integrate consumption concept into the freshmen education as soon as first-year students enter universities. We should promote various state and school funding policies and build a line of defense against harmful campus loans. In addition, we should pay close attention to the behavioral dynamics of students who have taken out campus loan, and guide them to consume reasonably and repay their debt in time to protect their personal credit score. There are several limitations in our study. In this manuscript, campus loan behavior is measured by binary variable. Therefore, whether college student has ever taken campus loans cannot measure the loan strength and sources. Therefore, these existing shortcomings need to be improved in the future. In the following research, we will construct technical route of joint control and prevention of campus loan control and real-time warning mechanism relying on big data.

## Data availability statement

The original contributions presented in this study are included in the article/supplementary material, further inquiries can be directed to the corresponding author/s.

## Ethics statement

Ethical review and approval was not required for the study on human participants in accordance with the local legislation and institutional requirements. Written informed consent from the patients/participants or patients/participants legal guardian/next of kin was not required to participate in this study in accordance with the national legislation and the institutional requirements.

## Author contributions

QZ and MZ: conceptualization and investigation. QZ: data curation, funding acquisition, and project administration. ZC: formal analysis. ZC and YZ: software. MZ: writing – review and editing. All authors: methodology, writing – original draft, and have read and agreed to the published version of the manuscript.
